# A multivariate predictive modeling approach reveals a novel CSF peptide signature for both Alzheimer's Disease state classification and for predicting future disease progression

**DOI:** 10.1371/journal.pone.0182098

**Published:** 2017-08-03

**Authors:** Daniel A. Llano, Saurabh Bundela, Raksha A. Mudar, Viswanath Devanarayan, for the Alzheimer’s Disease Neuroimaging Initiative (ADNI)

**Affiliations:** 1 Department of Molecular and Integrative Physiology, University of Illinois at Urbana-Champaign, United States of America; 2 Exploratory Statistics, AbbVie, Inc., North Chicago, IL, United States of America; 3 Department of Speech and Hearing Science, University of Illinois at Urbana-Champaign, United States of America; Banner Alzheimer's Institute, UNITED STATES

## Abstract

To determine if a multi-analyte cerebrospinal fluid (CSF) peptide signature can be used to differentiate Alzheimer’s Disease (AD) and normal aged controls (NL), and to determine if this signature can also predict progression from mild cognitive impairment (MCI) to AD, analysis of CSF samples was done on the Alzheimer’s Disease Neuroimaging Initiative (ADNI) dataset. The profiles of 320 peptides from baseline CSF samples of 287 subjects over a 3–6 year period were analyzed. As expected, the peptide most able to differentiate between AD vs. NL was found to be Apolipoprotein E. Other peptides, some of which are not classically associated with AD, such as heart fatty acid binding protein, and the neuronal pentraxin receptor, also differentiated disease states. A sixteen-analyte signature was identified which differentiated AD vs. NL with an area under the receiver operating characteristic curve of 0.89, which was better than any combination of amyloid beta (1–42), tau, and phospho-181 tau. This same signature, when applied to a new and independent data set, also strongly predicted both probability and rate of future progression of MCI subjects to AD, better than traditional markers. These data suggest that multivariate peptide signatures from CSF predict MCI to AD progression, and point to potentially new roles for certain proteins not typically associated with AD.

## Introduction

CSF biomarkers have been examined for their capacity to classify Alzheimer’s Disease (AD) disease state since they reflect the biochemical changes that occur in the AD brain. Three CSF biomarkers in particular, total tau (t-tau), phosphorylated tau (p-tau) and amyloid beta 42 (Aβ42), are believed to have high diagnostic accuracy for early AD diagnosis and have been used as research criteria for the diagnosis of AD [1–3]. A number of studies have found significantly reduced CSF Aβ42 levels in AD patients compared to normal controls with few exceptions (See [3] and [4] for meta-review and meta-analyses respectively; see [5,6] for exceptions). In comparison to Aβ42 levels, studies have consistently found increased CSF t-tau and p-tau levels in AD patients compared to normal controls (See [7] and [4] for meta-review and meta-analyses respectively). Furthermore, elevated levels of t-tau and p-tau have also been observed in MCI patients that developed AD compared to stable MCI patients and normal controls [8,9]. Given the possibility that a variety of other pathological processes may be simultaneously ongoing in the AD brain (e.g., oxidative stress, inflammation and synaptic dysfunction), apart from these three core CSF biomarkers, other biomarkers could reflect pathogenesis of AD and reveal new biomarkers for AD [10].

Proteomic approaches permit large-scale assessment of the involvement of hundreds of proteins and/or peptides in complex biological processes, and may generate hypotheses both about disease mechanisms and potential therapeutic targets. This type of approach has been used extensively to develop biomarkers and shape development of scientific hypotheses in the cancer literature [11–13]. One challenge to investigators utilizing proteomic approaches is the sheer mass of data that are obtained using these methods, both in terms of extraction of coherent trends in the data and in terms of the potential for spurious associations identified via multiple comparisons. We and others have addressed these potential problems by using machine learning algorithms to develop peptide “signatures” corresponding to disease state, and by employing strict criteria to avoid the potential for false discovery [14–18]. Increasingly, proteolytic fragments, rather than whole proteins, are being used for disease classification because of the expansion in the complexity of the signatures available [19–22]. Therefore, in the current report we explore the use of a proteomic technique applied to proteolytic fragments in the CSF for the classification and prediction of disease progression in AD.

Protein profiling of the CSF using advanced proteomics techniques such as 2D gel electrophoresis, mass spectrometry, and liquid chromatography-mass spectrometry could help identify novel AD biomarkers. While studies using proteomics techniques have identified a number of additional AD candidates (e.g., neuronal pentraxin receptor (NPTXR) and heart-type fatty acid binding protein (FABPH) [23–28]), many of these studies have been done on small cohorts [25,29] involving small arrays of CSF markers, using less powerful computational approaches and did not validate the markers in an independent cohort. To circumvent these issues, we performed cross sectional analysis of CSF samples obtained from large and well characterized populations of AD, MCI, and age-matched normal control (NL) subjects from the Alzheimer’s Disease Neuroimaging Initiative (ADNI) study. We analyzed a diverse array of peptides to determine if single or multi-analyte CSF peptide signatures could be used to (i) distinguish patients with AD from NL (disease state classification) and (ii) predict future conversion from MCI to AD in a separate population of patients (prediction of future progression).

## Methods

Data were obtained from the ADNI database (adni.loni.usc.edu). ADNI was launched in 2003 as a public-private partnership, led by Principal Investigator Michael W. Weiner, MD. The primary goal of ADNI has been to test whether serial magnetic resonance imaging (MRI), positron emission tomography (PET), other biological markers, and clinical and neuropsychological assessments can be combined to measure the progression of MCI and early AD. For up-to-date information, see www.adni-info.org. This study was conducted across multiple clinical sites and was approved by the Institutional Review Boards of all of the participating institutions. Informed written consent was obtained from all participants at each site. The following individual ethics boards approved the study: Albany Medical College Institutional Review Board, Boston University Medical Campus Institutional Review Board (BU IRB), Butler Hospital Institutional Review Board, Cleveland Clinic Institutional Review Board, Columbia University Institutional Review Board, Dartmouth-Hitchcock Medical Center Committee for the Protection of Human Subjects, Duke University Health System Institutional Review Board, Emory University Institutional Review Board Georgetown University Institutional Review Board, Human Investigation Committee Yale University School of Medicine, Human Subjects Committee, University of Kansas Medical Center, Indiana University Institutional Review Board, Research Compliance Administration, Institutional Review Board of Baylor College of Medicine, Institutional Review Board of the Mount Sinai School of Medicine, Johns Hopkins University School of Medicine Institutional Review Boards, Lifespan—Rhode Island Hospital Institutional Review Board, Mayo Clinic Institutional Review Board, Nathan Kline Institute Rockland Psychiatric Center Institutional Review Board (NKI RPC IRB), New York University Langone Medical Center School of Medicine, Institutional Review Board Human Research Program, Northwestern University Institutional Review Board Office, Office of the Washington University School of Medicine IRB (OWUMC IRB), Oregon Health and Science University Institutional Review Board, Partners Human Research Committee, Research Ethics Board Jewish General Hospital, Research Ethics Board Sunnybrook Health Sciences Centre, Roper St. Francis Institutional Review Board, Rush University Medical Center Institutional Review Board, Stanford University, Administrative Panel on Human Subjects in Medical Research, The Ohio State University Institutional Review Board, The University of Texas Southwestern Medical Center Institutional Review Board, UCLA Office of the Human Research Protection Program Institutional Review Board, UCSD Human Research Protections Program, University Hospitals Case Medical Center Institutional Review Board, University of Alabama at Birmingham Institutional Review Board, University of British Columbia, Clinical Research Ethics Board (CREB), University of California Davis Office of Research IRB Administration, University of California Irvine Office Of Research Institutional Review Board (IRB), University of California San Francisco Committee on Human Research (CHR), University of Iowa Institutional Review Board, University of Kentucky Office of Research Integrity, University of Michigan Medical School Institutional Review Board (IRBMED), University of Pennsylvania Institutional Review Board, University of Pittsburgh Institutional Review Board, University of Rochester Research Subjects Review Board (RSRB), University of South Florida Division of Research Integrity & Compliance, University of Southern California Health Science Campus Institutional Review Board, University of Western Ontario Research Ethics Board for Health Sciences Research Involving Human Subjects (HSREB), University of Wisconsin Health Sciences Institutional Review Board, Wake Forest University Institutional Review Board, Weill Cornell Medical College Institutional Review Board, Western Institutional Review Board and Western University Health Sciences Research Ethics Board.

### Patient population

Participants included patients with AD (defined by NINCDS-ADRDA1) and MCI (using Petersen criteria [30]), and NL from the ADNI study that received clinical, neuropsychological, and biomarker assessments which were repeated every 6 months for up to 36 months. NL individuals were free of memory complaints or depression and had a Mini-Mental State Examination (MMSE) score above 25 and a Clinical Dementia Rating (CDR) score of 0. MCI individuals could have MMSE scores of 23 to 30 and required a CDR of 0.5 and an informant-verified memory complaint substantiated by abnormal education-adjusted scores on the Wechsler Memory Scale Revised—Logical Memory II. AD patients could have MMSE scores of 20 to 27 and a CDR of 0.5 or 1.0. Of the 135 MCI subjects from whom the CSF proteomic data were available at baseline, 122 subjects stayed in the study for at least 36 months.

### CSF samples

CSF samples (0.5 mL) were obtained in the morning after an overnight fast and processed using the Caprion Proteomics platform that uses mass spectrometry to evaluate the ability of a panel of peptides to discriminate disease states and disease progression. Procedures for CSF sampling, transport, and storage have been described previously [31]. The CSF multiplex multiple reaction monitoring (MRM) panel was developed by Caprion Proteomics in collaboration with the Biomarker Consortium Project Team. A total of 320 peptides generated from tryptic digests of 143 proteins were used in this study (see S1 Table for list of peptides and proteins). These peptides include a series of peptides representing inflammatory markers and peptides identified in an earlier phase of the program that used multiplexed immunoassay based platform (performed by Rules Based Medicine).

Details regarding the technology, quality control and validation can be found in the Use of Targeted Mass Spectrometry Proteomic Strategies to Identify CSF-Based Biomarkers in Alzheimer’s Disease Data Primer (http://adni.bitbucket.org/csfmrm.html). In brief, as described in the data primer and in Spellman et al. (2015) [32], CSF samples were depleted of plasma proteins using a Multiple Affinity Removal System (MARS-14) column, trypsin digested (1:25 protease:protein ratio), lyophilized, desalted and analyzed by LC/MRM-MS analysis on a QTRAP 5500 LC-MS/MS system at Caprion Proteomics. MRM is a mass spectrometry-based platform that has been shown to be reproducible within and across laboratories and instrument platforms [33]. MRM experiments were performed on triple quadrupole (Q) mass spectrometers. The first (Q1) and third (Q3) mass analyzer were used to isolate a peptide ion and a corresponding fragment ion. The fragment ions were generated in Q2 by collision induced dissociation (CID). The 320 peptides met all the quality control criteria set by the ADNI working group.

### Analysis

For the univariate analysis to identify individual peptides that are either differentially expressed between AD and NL subjects, or between MCI-AD progressors versus non-progressors, the analysis of covariance model (ANCOVA) was used with age and gender as covariates and the groups to be compared as fixed effect. This model was fit on the log2 transformed quantile-normalized intensities of the peptide expression values. Outliers were identified and excluded based on the residuals from this ANCOVA model whose values were either less than Q1–1.5 x (Q3-Q1) or above Q1 + 1.5 x (Q3-Q1), where Q1 and Q3 are the first and third quartiles of the distribution of residuals. The significance of peptides was assessed and is reported in terms of the false discovery rate estimate (q-value) [34], and the relevant summary statistics such as the receiver operator characteristic area under the curve (ROC AUC), fold change, and the effect size, along with p-values are also reported.

Multivariate predictive modeling analysis was then carried out to derive a signature (combination of peptides and any additional covariates) that optimally differentiates the AD versus NL subjects. The list of candidate predictors considered for selection in this signature included the list of 320 peptides of the CSF proteomic panel, plus age, gender and apolipoprotein E (APO-E) status (totally 323 predictors). An algorithm based on the logistic regression model with lasso-based penalty [35] was employed for this analysis. To ensure the stability and robustness of the selection of a subset of predictors for the optimal signature via this algorithm, a bootstrap procedure [36] was used to estimate the lasso penalty parameter. The performance of the optimal peptide signature from this algorithm that differentiates the AD and NL subjects was evaluated via a rigorous five-fold internal stratified cross-validation procedure. In this procedure, all steps of the model building and signature derivation process were fully embedded within the cross-validation [37]. The predictions of all the left-out folds from this cross-validation procedure [14] were first aggregated, and the performance measures such as the overall classification accuracy, sensitivity, specificity, and the positive and negative predictive values were evaluated on these aggregated predictions. This internal cross-validation procedure was repeated 20 times, and the mean and standard deviation of these performance measures are reported.

The above optimal peptide signature derived to differentiate the AD and NL subjects was then tested on a separate independent group of MCI subjects at baseline to predict their future progression to AD. As the peptide signature would return the prediction results as simply AD or NL, the prediction of an MCI subject as NL was considered as “Signature Negative” at baseline, and the prediction of an MCI subject as AD was considered as “Signature Positive” at baseline. The accuracy of this prediction was then assessed relative to the true progression status of the MCI subjects to AD over the next 36 months.

The performance of this peptide signature was further evaluated in terms of its ability to differentiate the future “time to progression” from MCI to AD of these baseline signature positive and signature negative MCI subjects via Kaplan-Meier analysis. For this evaluation, the progression of MCI subjects to AD over the entire future time course until the last follow-up visit was taken into consideration. This evaluation of the AD versus NL peptide signature on the future progression of a separate group of MCI subjects to AD would not only serve as an independent verification of the utility of our peptide signature, but also put it to a greater test to see whether it is robust enough to address a different and more important question related to predicting the future disease progression in AD.

## Results

### Disease-state demographics

Data from 287 subjects were analyzed, with the largest proportion (135/287 or 47.1%) coming from MCI subjects. Of the 66 AD subjects, 65 were diagnosed as “probable” and 1 was diagnosed as “possible” AD. The subjects were balanced across the NL, MCI and AD groups in terms of age (range of means = 74.79–75.80 years, p>0.05) and education (range of means = 15.11–16.0 years, p>0.05). There were more males (59.9%) than females (40.1%) in the study, though similar numbers of male and female MCI subjects converted to AD over a three-year period (52.3 vs. 65.8%, p = 0.166, Chi-squared test). As shown previously [38], the presence of the APO-E4 allele tracked with disease state (71.2% AD, 52.6% MCI and 31.8% NL, p < 0.0001, Chi-squared test). In addition, the presence of this allele also tracked with MCI to AD progression over a 36-month period (37.5% of non-E4 vs. 56.3% of E4 progressed to AD, p = 0.028, Chi-squared test), see Tables 1 and 2.

**Table 1:** Disease-state demographics.

		AD (n = 66)	MCI (n = 135)	NL (n = 86)
**Gender (n)**	M	37	91	44
	F	29	44	42
**Apo-E (n)**	E4	47	71	21
	Non-E4	19	64	65
**Age** **(years, mean +/- SD)**		75.09 ± 7.52	74.79 ± 7.36	75.80 ± 5.55
**Education** **(years, mean +/- SD)**		15.11 ± 2.96	16 ± 3	15.64 ± 2.97
**Baseline MMSE** **(mean +/- SD)**		23.52 ± 1.85	26.91 ± 1.74	29.05 ± 1.02

**Table 2:** Three-year MCI converter vs. nonconverter demographics.

		MCI to AD converters (n = 64)	MCI non-converters (n = 71)
**Gender (n)**	M	**40**	**51**
	F	**24**	**20**
**Apo-E (n)**	E4	**40**	**31**
	Non-E4	**24**	**40**
**Age (years, mean +/- SD)**		**74.92 +/- 7.57**	**74.68 +/- 7.21**
**Education (years, mean +/- SD)**		**15.59 +/- 3.02**	**16.36 +/- 2.89**
**Baseline MMSE (mean +/- SD)**		**26.36 +/- 1.68**	**27.41 +/- 1.64**

### Disease-state classification: Univariate analysis

**Fig 1:**
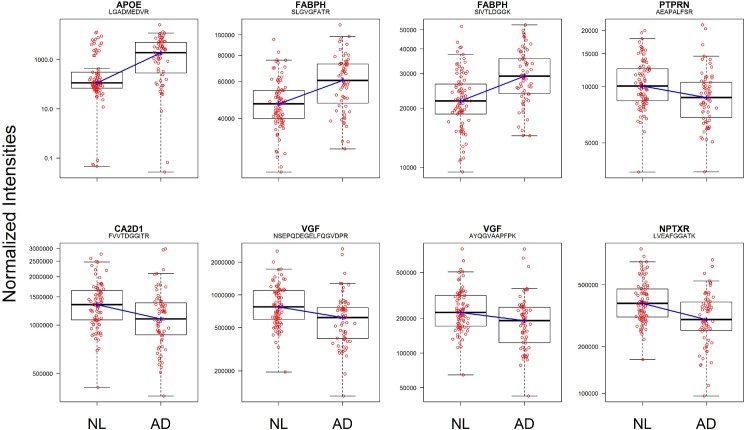
Univariate analysis.Values of the 8 peptide markers with the lowest q-values in AD vs. NL disease state classification. Individual subjects are shown as open circles. Boxes represent the first and third quartiles. The lines that extend out from the top and bottom ends of box indicate the range of the range, minus the outliers. The points outside the lines are the low and high outliers.

A large number of peptides were found to be differentially present in AD vs. NL subjects. As expected, one APO-E peptide sequence was present in substantially higher amounts in AD vs. NL subjects (APOE_LGADMEDVR: 17.29 fold difference in median value, q = 9.45E-07, see Table 3). This finding was previously known since this sequence is found only in APOE4+ subjects [39,40]. Other peptides, some known to be involved in neuronal function (e.g., CA2D1, the voltage-dependent calcium channel subunit alpha-2/delta-1), and others not classically associated with neuronal function (e.g., FABPH), differed between AD and NL subjects. Using a q-value < 0.05 criteria, 39 out of 320 peptides reached statistical significance with this false discovery rate correction, while 11 out of 320 had q-values less than 0.005 (see Fig 1 for the top 8 peptides).

**Table 3:** Normal vs. Alzheimer Disease, univariate analysis. Shown are the analytes with a q-value < 0.05.

Symbol	Sequence	Fold Change	ROC AUC	Effect size	p-value	q-value
APOE	LGADMEDVR	17.29	0.73	0.74	2.95E-09	9.45E-07
FABPH	SLGVGFATR	1.30	0.72	0.81	1.57E-08	2.51E-06
FABPH	SIVTLDGGK	1.35	0.73	0.86	2.49E-07	2.66E-05
PTPRN	AEAPALFSR	0.87	0.66	-0.54	1.18E-05	0.0009
CA2D1	FVVTDGGITR	0.82	0.65	-0.55	3.14E-05	0.0019
VGF	NSEPQDEGELFQGVDPR	0.80	0.67	-0.62	3.52E-05	0.0019
VGF	AYQGVAAPFPK	0.83	0.64	-0.53	4.72E-05	0.0022
NPTXR	LVEAFGGATK	0.78	0.69	-0.73	6.15E-05	0.0025
CCKN	AHLGALLAR	0.82	0.65	-0.47	8.84E-05	0.0031
PTPRN	SELEAQTGLQILQTGVGQR	0.88	0.63	-0.50	9.83E-05	0.0031
NPTXR	ELDVLQGR	0.84	0.69	-0.70	1.34E-04	0.0039
PIMT	VQLVVGDGR	0.86	0.66	-0.58	0.0003	0.0070
SCG1	NYLNYGEEGAPGK	0.82	0.67	-0.60	0.0003	0.0070
SCG2	VLEYLNQEK	0.91	0.63	-0.44	0.0004	0.0097
CH3L1	ILGQQVPYATK	1.09	0.62	0.48	0.0005	0.0100
VGF	THLGEALAPLSK	0.88	0.63	-0.50	0.0005	0.0104
FAM3C	GINVALANGK	0.86	0.63	-0.44	0.0006	0.0108
AMD	IVQFSPSGK	0.85	0.66	-0.57	0.0006	0.0108
AMD	IPVDEEAFVIDFKPR	0.90	0.62	-0.40	0.0007	0.0121
CA2D1	TASGVNQLVDIYEK	0.88	0.65	-0.46	0.0008	0.0121
CA2D1	IKPVFIEDANFGR	0.85	0.63	-0.44	0.0008	0.0121
CMGA	SEALAVDGAGKPGAEEAQDPEGK	0.87	0.64	-0.43	0.0009	0.0137
CMGA	YPGPQAEGDSEGLSQGLVDR	0.82	0.62	-0.38	0.0010	0.0145
NEGR1	SSIIFAGGDK	0.91	0.63	-0.51	0.0012	0.0155
CH3L1	SFTLASSETGVGAPISGPGIPGR	1.08	0.60	0.43	0.0013	0.0155
SCG1	HLEEPGETQNAFLNER	0.81	0.62	0.02	0.0013	0.0155
CMGA	EDSLEAGLPLQVR	0.77	0.63	-0.25	0.0013	0.0155
NPTX2	LESLEHQLR	0.81	0.64	-0.52	0.0014	0.0156
NRCAM	VFNTPEGVPSAPSSLK	0.89	0.64	-0.49	0.0016	0.0173
FAM3C	SPFEQHIK	0.95	0.61	-0.44	0.0021	0.0225
PCSK1	GEAAGAVQELAR	0.87	0.63	-0.49	0.0024	0.0252
NPTX1	LENLEQYSR	0.89	0.63	-0.53	0.0026	0.0255
PCSK1	ALAHLLEAER	0.85	0.63	-0.51	0.0032	0.0308
SCG3	FQDDPDGLHQLDGTPLTAEDIVHK	0.84	0.63	-0.42	0.0035	0.0331
NPTX2	TESTLNALLQR	0.86	0.64	-0.55	0.0037	0.0341
TTHY	TSESGELHGLTTEEEFVEGIYK	1.08	0.62	0.39	0.0051	0.0445
PDYN	LSGSFLK	0.87	0.61	-0.35	0.0052	0.0445
PCSK1	NSDPALGLDDDPDAPAAQLAR	0.86	0.63	-0.44	0.0055	0.0464
NRCAM	YIVSGTPTFVPYLIK	0.89	0.60	-0.32	0.0057	0.0468

### Disease-state classification: Multivariate analysis

**Fig 2:**
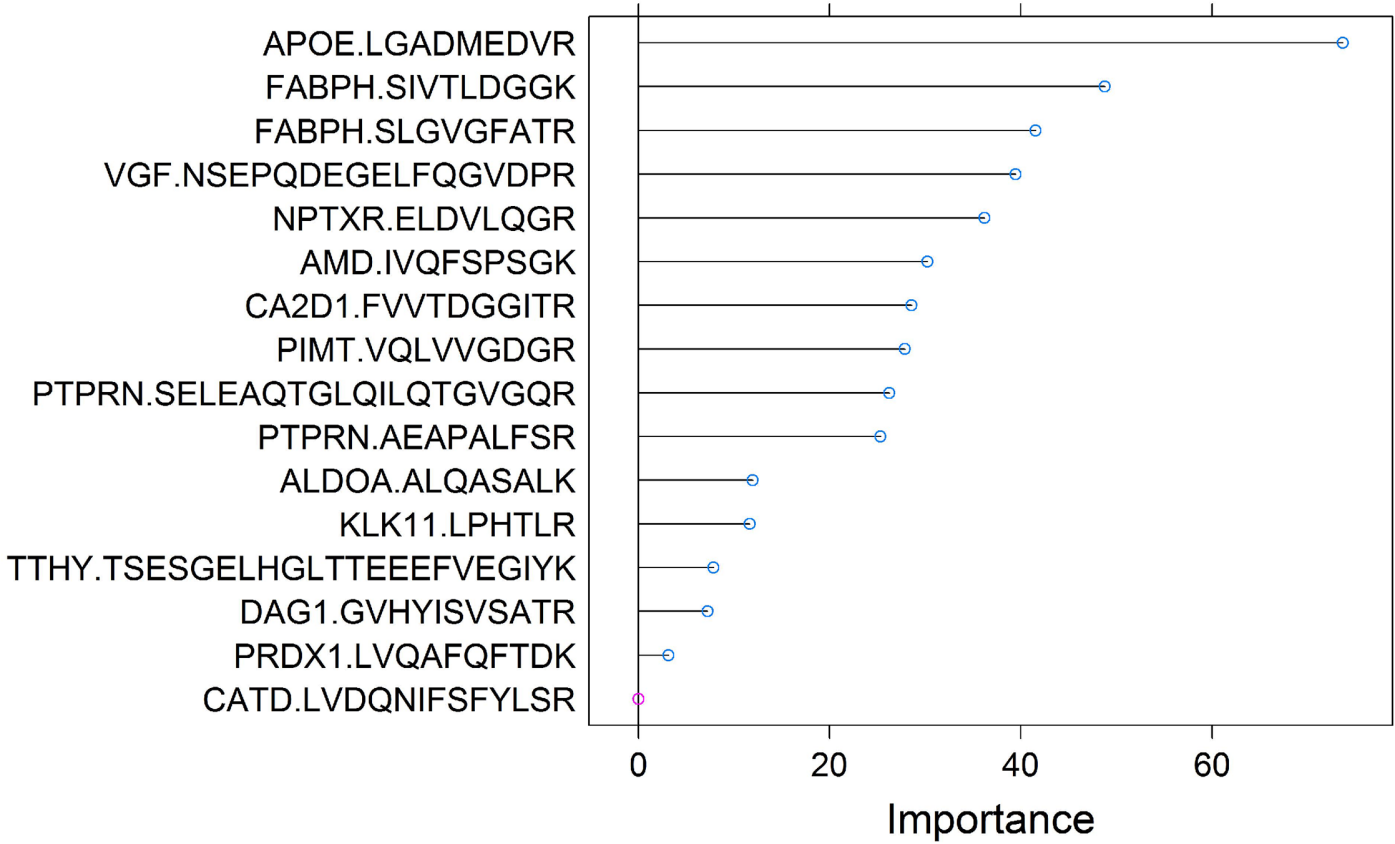
16-peptide signature. Relative importance of the contribution of each peptide in the 16-peptide multivariate signature for differentiating AD vs. NL that is subsequently used for predicting progression of MCI subjects to AD. Peptides are plotted in the order of their importance/contribution to this multivariate signature in the logistic regression model. As the 16^th^ peptide related to CATD appears to provide very little incremental value (noted in red), the data-driven process that led to its inclusion in the signature suggested an overall benefit of retaining it in the signature. The coefficients for each of these markers is given in S2 Table.

Creation of an optimized multivariate signature improved disease state differentiation compared to individual peptides. Inclusion of all 320 peptide sequences, demographic data (age, gender, education) and APO-E4 status produced an optimized 16-peptide signature. The size of our signatures and contents were determined via a totally data-driven manner via the mathematical optimization and algorithm described in the Methods section in detail. The signature components are shown in Fig 2, coefficients are shown in S2 Table. Though this model’s ability to differentiate AD from NL was relatively modest, with the area under its receiver-operating characteristic curve (ROC AUC) of 0.89 +/- 0.01 (based on 20 iterations of 5 fold cross validation), this value was higher than that seen of any individual marker (highest was APO-E with 0.73).

The performance of the 16-peptide multivariate signature was compared to all permutations of Aβ42, t-tau and p-tau (181) in the CSF, including their ratios, and published cut-points [17]. Across all measures, the 16-peptide multivariate signature outperformed the other markers significantly (Table 4). In addition, including Aβ42, t-tau and p-tau (181) with the 16-peptide signature did not result in a significant improvement in performance.

**Table 4:** Performance of multivariate model to differentiate disease state. The top row corresponds to all permutations of Ab, tTau and pTau, and the bottom row refers to the 16-peptide signature shown in Fig 2.

	Accuracy	Sensitivity	Specificity	PPV	NPV
**Aβ**_**1–42**_**, tTau, pTau signature**	0.78 +/- 0.01	0.80 +/- 0.02	0.75 +/- 0.02	0.71 +/- 0.01	0.84 +/- 0.01
**16-peptide signature**	0.85 +/- 0.02	0.86 +/- 0.02	0.84 +/- 0.03	0.80 +/- 0.03	0.89 +/- 0.01

### MCI-AD progression: Univariate analysis

We compared CSF profiles for MCI patients that converted to AD by the 36 month visit vs. MCI patients that did not convert. Three markers had marginal q-values of 0.0508: hemoglobin subunit alpha (HBA), neuronal pentraxin 2 (NPTX2) and poliovirus receptor-related protein 1 (PVRL1, Table 5). Interestingly, the APO-E peptide (LGADMEDVR), which demonstrated excellent differentiation between AD vs. NL, ranked 199/320 for predicting conversion from MCI to AD. These data suggest that individual peptide markers do a poor job of predicting MCI to AD progression on their own; hence the motivation to combine markers in a multivariate analysis to increase their utility (below).

**Table 5:** MCI to AD converters vs. non-converters, univariate analysis, lowest 20 q-values.

Symbol	Sequence	Fold Change	ROC AUC	Effect size	p-value	q-value
HBA	FLASVSTVLTSK	1.66	0.63	0.47	0.0006	0.19
NPTX2	LESLEHQLR	0.80	0.65	-0.52	0.0013	0.19
HBA	VGAHAGEYGAEALER	2.68	0.64	0.49	0.0017	0.19
HBB	SAVTALWGK	2.23	0.63	0.44	0.0046	0.28
HBB	VNVDEVGGEALGR	2.11	0.63	0.46	0.0049	0.28
PRDX1	DISLSDYK	1.12	0.61	0.36	0.0061	0.28
NPTX2	TESTLNALLQR	0.71	0.63	-0.47	0.0070	0.28
NRCAM	SLPSEASEQYLTK	0.90	0.59	-0.34	0.0071	0.28
HBA	TYFPHFDLSHGSAQVK	1.46	0.61	0.44	0.0128	0.37
CO3	IHWESASLLR	0.55	0.64	-0.41	0.0133	0.37
CFAB	VSEADSSNADWVTK	0.88	0.63	-0.45	0.0137	0.37
HBB	EFTPPVQAAYQK	2.18	0.61	0.52	0.0138	0.37
PVRL1	ITQVTWQK	0.92	0.63	-0.45	0.0164	0.40
CFAB	YGLVTYATYPK	0.84	0.60	-0.36	0.0222	0.42
CO2	HAIILLTDGK	0.92	0.60	-0.37	0.0227	0.42
NPTXR	ELDVLQGR	0.85	0.61	-0.39	0.0245	0.42
CAH1	YSSLAEAASK	1.35	0.59	0.31	0.0275	0.42
C1QB	VPGLYYFTYHASSR	0.92	0.59	-0.26	0.0284	0.42
TTHY	VEIDTK	1.10	0.59	0.24	0.0287	0.42
PRDX6	LSILYPATTGR	1.29	0.59	0.23	0.0287	0.42

### MCI-AD progression: Multivariate analysis

**Fig 3:**
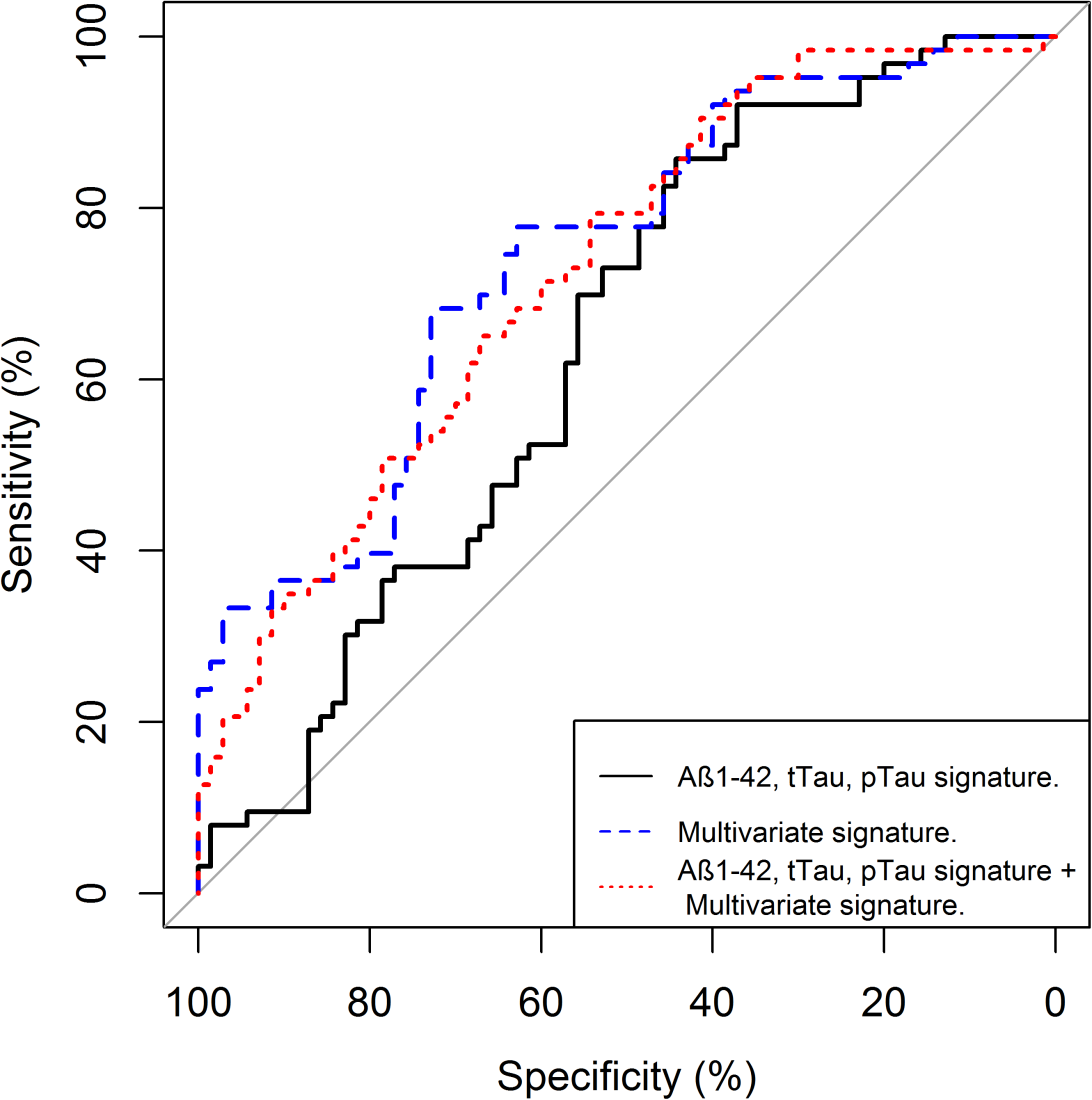
Receiver-operator curves. Receiver-operator curves comparing the 16-peptide multivariate signature (red dotted line) to combinations of Aβ42, tau and p-tau 181 (black line) as well as the 16-peptide signature + combinations of Aβ42, tau and p-tau 181 (blue dashed line) for the prediction of 36-month conversion from MCI to AD.

The same 16-peptide multivariate signature that was developed for disease state classification was employed on the MCI subjects, which represent a completely independent population, at baseline to predict their progression to AD over 36 months. As shown in Table 6, across all measures, the 16-peptide signature outperformed all permutations of Aβ42, t-tau and p-tau (181) and published cut-points [17]. Receiver-operator curves were constructed using all combinations of markers for Aβ42 and different forms of tau, for the 16-peptide signature shown in Fig 2 and for a combination of the two for predicting the 36-month MCI-AD progression. The largest area under the curve was observed for the 16-peptide signature (0.74), with a similar value seen for the combined 16-peptide + Aβ42/tau markers (0.73) and the lowest seen for combinations of Aβ42/tau markers without the multivariate signature (0.64, p < 0.05, Fig 3).

**Table 6:** Performance of multivariate model to differentiate MCI to AD converters vs. non-converters. The top row corresponds to all permutations of Ab, tTau and pTau, and the bottom row refers to the 16-peptide signature shown in Fig 2.

	Accuracy	Sensitivity	Specificity	PPV	NPV
**Aβ**_**1–42**_**, tTau, pTau signature**	0.62	0.78	0.49	0.58	0.71
**16-peptide signature**	0.70	0.78	0.63	0.65	0.76

**Fig 4:**
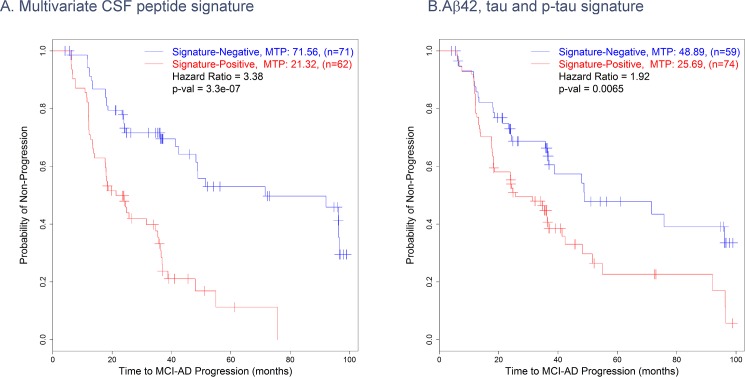
Kaplan-Meier curves. A) Kaplan-Meier curves over the entire time course until the last follow-up visit that show the relative rates of future progression to AD for the MCI subjects identified as signature positive or negative at baseline by the 16-peptide multivariate signature. B) Similar curves, but for subjects that were identified as signature positive or negative by the Aβ/t-Tau/p-Tau biomarkers. MTP = mean time to progression.

The 16-peptide AD vs NL multivariate signature was then tested on the MCI subjects at baseline to predict their progression to AD over the entire future time course up to the last follow-up visit. The classifier built based on the 16-peptide AD vs NL signature was used to place the MCI patients at baseline into two categories; those predicted as NL were considered as “Signature Negative” and those predicted as AD were considered as “Signature Positive”. As evident from Fig 4A, MCI subjects in the signature positive group at baseline had a much faster median time to progression (MTP) to AD than those in the signature negative group (21.32 months versus 71.56 months, p = 3.3 x 10^−7^, hazard ratio = 3.38). While similar analysis using combinations of Aβ42, t-tau and p-tau (181) to place the MCI subjects into signature positive and negative groups at baseline reveal faster progression of the signature positive MCI subjects to AD (MTP of 25.69 versus 48.89 months, p = 0.0065, hazard ratio = 1.92, Fig 4B), the 16-peptide signature provided a more robust predictor of MTP (Table 7). The 16-peptide signature also outperformed the published cut-points on Aβ42, t-tau and p-tau (181) [17], which had a hazard ratio of 1.8. These data suggest that 16-peptide signature is a strong predictor of future progression from MCI to AD over the subsequent years and outperforms the traditional CSF biomarkers.

**Table 7:** Performance of AD vs. NL multivariate signatures to differentiate the Time to Progression of MCI subjects to AD.

	Median Time to MCI-AD Progression		Hazard Ratio	p-value
	Signature Negative	Signature Positive		
**Aβ**_**1–42**_**, tTau, pTau signature**	48.89	25.69	1.92	0.0065
**16-peptide signature**	71.56	21.32	3.38	3.3 x 10^−7^

## Discussion

In this study, the diagnostic and predictive accuracy of an array of 300+ peptides in the CSF for the diagnosis of MCI and AD and for the prediction of progression from MCI to AD was examined. It was found that several individual peptides, including many not classically associated with neuronal function, showed high statistical significance in distinguishing between AD and NL. A 16-peptide multivariate signature based on these peptides was identified with an overall classification accuracy of 85%, with improved accuracy, sensitivity, specificity and positive and negative predictive values compared to more traditional CSF markers. More notably, when this same 16-peptide signature was tested on an independent group of 135 MCI subjects, it outperformed the traditional Aβ/tau markers for predicting the future progression from MCI to AD; a positive result on this 16-peptide multivariate signature at baseline resulted in a 3.38-fold faster progression to AD. Though some of these peptides have been described previously as individual biomarkers (see below), the current data suggest their combination outperforms previous CSF markers and point to the possibility that other novel markers may have a previously unrecognized role in diagnostic testing as well as in understanding the pathophysiology of AD.

### Review of specific analytes identified

Over the past several years, proteomic approaches have identified an alphabet soup of potential markers that may be able to permit early diagnosis of AD or predict conversion from MCI to AD [24–26,41–45]. Many of the potential markers identified by these studies have known or suspected roles in either AD or in pathological processes thought to be disrupted in AD. For example, as expected, one of the APOE peptides examined (LGADMEDVR), which is specifically expressed in APOE4+ individuals [39,40], showed different distributions between AD and NL subjects (Table 3). This finding is not surprising given APOE4’s known association with AD [38].

Other peptides identified in this study are less classically associated with AD. For example, we observed that CSF FABPH levels were elevated in AD relative to controls and this marker has previously been identified by several studies as being associated with AD [25,46–50]. FABPH is a small cytoplasmic protein involved in lipid metabolism and was initially identified as a potential biomarker for cardiac injury [51] but also present in neurons [52]. It is not clear if the presence of FABPH in the CSF is a marker of neuronal dysfunction, or simply a marker of neuronal destruction, since elevations of serum and/or CSF FABPH have been seen in Creutzfeldt–Jakob disease, traumatic brain injury, ischemic stroke and subarachnoid hemorrhage [53–57]. These findings may imply that FABPH is released from neurons during their destruction, or alternatively that high CSF FABPH may predispose neurons to be vulnerable to oxidative stress, since overexpression of FABPH sensitizes dopaminergic neurons to the toxic effects of metabolic stressors such as 1-methyl-4-phenylpyridine, (MPP), while low levels of FABPH are protective [58]. For these reasons, FABPH has been proposed by others to serve as a general biomarker for synaptic destruction [57], analogous to creatine kinase or troponin for myocyte damage.

We also identified NPTXR as being associated with AD, with CSF levels being lower in AD compared to controls and higher in MCI-AD converters. These data, combined with previous work demonstrating that NPTXR levels may be slightly higher in MCI subjects than controls, but drop rapidly (by ~10%/year) in AD subjects, suggest that NPTXR is a dynamic biomarker [26]. The current data are also consistent with work in presymptomatic subjects carrying PSEN1 or the APP genetic mutations, who show elevated levels of CSF NPTXR [45]. The transient increases, then prominent drops, in CSF NPTXR levels suggest a complex relationship between NPTXR levels and disease state, and are reminiscent of what is seen with other AD biomarkers. For example, we previously observed pseudonormalization of several plasma biomarkers that appeared to have a transition state in MCI patients that differed from controls, then returned to baseline in AD [14]. Analogously, hippocampal blood-oxygen level dependent signals increase in MCI subjects compared to NL, but then decrease in AD subjects [59]. The complex relationship between NPTXR and disease state is evidenced by some of the conflicting data in the literature. At least two other studies have found decreases in CSF NPTXR in AD [23,26] while one documented higher levels of NPTXR in the CSF of patients with MCI and AD [60]. The severity of cognitive impairment of the AD patients was not given in the latter study, and it is possible that the AD patients in their study were too early in their course to show a drop in NPTXR levels. Alternatively, the method of measurement (Western blot) differs compared to the current study and may account for the discrepancy.

Additional markers were revealed in both the univariate and multivariate analyses. For example, neurosecretory protein VGF (VGF), a nerve growth factor-responsive molecule which is likely a precursor to several bioactive peptides, has been localized to the human and rat cerebral cortex, and in the current study was found to be lower in the CSF of AD patients (Fig 1) and ranked fourth in importance in multivariate signature (Fig 2). Several previous reports have found depressed levels of VGF in the brain [61] and CSF [62–66] of AD patients relative to controls, similar to the current findings. Additionally, CSF VGF levels are also diminished in acute pediatric encephalopathy [67] and frontotemporal dementia [68], suggesting that diminished CSF VGF may be a general marker of severe neuronal dysfunction. An additional novel finding here is the potential role of receptor-type tyrosine-protein phosphatase-like N (PTPRN) in AD, as revealed by both the univariate and multivariate analyses. PTPRN is a transmembrane protein implicated in multiple functions, including metabolism, growth and differentiation and is expressed in neurons [69]. Previous work has indicated that single nucleotide polymorphisms for the PTPRN gene were differentially related to CSF p-tau levels in an MCI-AD converter group compared to an MCI nonconverter group [69]. To our knowledge, this is the first report that PTPRN levels are depressed in the CSF of AD patients and, given PTPRN’s role in metabolism [70], may open vistas to further examine metabolic theories of the development of AD.

The current data also suggest a potential role for hemoglobin subunits in the prediction of conversion from MCI to AD. This could point to blood contamination, but in a recent analysis of this dataset, hemoglobin subunit levels were not found to correlate with CSF erythrocyte counts [32]. It is possible that these peptides represent blood-brain barrier breakdown, which has been documented to occur in AD [71], suggesting that this breakdown may be an early marker for MCI to AD conversion.

Finally, a recent study also examined the current dataset and proposed a multivariate signature to predict MCI to AD conversion [32]. The 29-peptide signature in the Spellman et al. study contained peptides from several proteins found in the signature from the current study (ALDOA, FABPH, NPTXR, PRDX1, VGF), while several peptides did not overlap. It is important to note that the signature observed in the Spellman et al. study was built and then tested on the same MCI-to-AD conversion dataset. In contrast, in the current study the signature was built on one dataset (AD vs. NL) and used to predict MCI-to-AD conversion on a completely independent group of subjects, increasing the external validity of the current approach. This methodological difference may explain these differences in peptide signatures.

## Conclusion

This study suggests that a novel signature of CSF peptides outperforms traditional CSF markers for the differentiation of AD from NL and prediction of future MCI to AD conversion. Note that similar accuracy in predicting AD conversion was seen in a similar analysis using multiple markers (APO-E genotype, neuropsychological testing and multiple imaging modalities [72]). However, it may be impractical to obtain all of these markers from individual patients. Therefore, one potential advantage of the current approach is that a single CSF study may be sufficient for prediction of progression. The current study also extends recent findings that FABPH and NPTXR may serve as CSF markers for the diagnosis of AD and prediction of disease progression. In addition, this work also highlights potentially novel biochemical pathways affected in AD and may help open new avenues of investigation to the underlying mechanisms of AD pathogenesis.

## Supporting information

S1 Table List of peptides. All peptides, proteins and UniProt accession numbers from the peptides measured in this study.
(DOCX)

S2 Table Signature coefficients. Coefficients for the signature peptides are given. Caution should be exercised in interpreting these coefficients: 1. Lower coefficient does not imply less importance because the scales of the intensity values are different between the peptides, and 2. These values should not be applied directly in practice unless the same exact assay platform is used.
(DOCX)
