# Supplementary material for: A multivariate predictive modeling approach reveals a novel CSF peptide signature for both Alzheimer's Disease state classification and for predicting future disease progression
Source: PLoS One. 2017 Aug 3;12(8):e0182098. doi: 10.1371/journal.pone.0182098 (PMC5542644; doi:10.1371/journal.pone.0182098)
Supplement: S1 Table — All peptides, proteins and UniProt accession numbers from the peptides measured in this study. (DOCX) [file pone.0182098.s001.docx]

S1 Table. List of Peptides. All peptides, proteins and UniProt accession numbers from the peptides measured in this study.

| **Peptide** | **Protein** | **Uniprot accession number** |
| --- | --- | --- |
| A1AT_AVLTIDEK | Alpha-1-antitrypsin | [P01009](http://www.uniprot.org/uniprot/P01009) |
| A1AT_LSITGTYDLK | Alpha-1-antitrypsin | [P01009](http://www.uniprot.org/uniprot/P01009) |
| A1AT_SVLGQLGITK | Alpha-1-antitrypsin | [P01009](http://www.uniprot.org/uniprot/P01009) |
| A1BG_NGVAQEPVHLDSPAIK | Alpha-1B-glycoprotein | [P04217](http://www.uniprot.org/uniprot/P04217) |
| A1BG_SGLSTGWTQLSK | Alpha-1B-glycoprotein | [P04217](http://www.uniprot.org/uniprot/P04217) |
| A2GL_DLLLPQPDLR | Leucine-rich alpha-2-glycoprotein | P02750 |
| A2GL_VAAGAFQGLR | Leucine-rich alpha-2-glycoprotein | P02750 |
| A4_LVFFAEDVGSNK | Amyloid beta A4 protein | P05067 |
| A4_THPHFVIPYR | Amyloid beta A4 protein | P05067 |
| A4_WYFDVTEGK | Amyloid beta A4 protein | P05067 |
| AACT_ADLSGITGAR | Alpha-1-antichymotrypsin | P01011 |
| AACT_EIGELYLPK | Alpha-1-antichymotrypsin | P01011 |
| AACT_NLAVSQVVHK | Alpha-1-antichymotrypsin | P01011 |
| AATC_IVASTLSNPELFEEWTGNVK | Aspartate aminotransferase, cytoplasmic | P17174 |
| AATC_LALGDDSPALK | Aspartate aminotransferase, cytoplasmic | P17174 |
| AATC_NLDYVATSIHEAVTK | Aspartate aminotransferase, cytoplasmic | P17174 |
| AATM_FVTVQTISGTGALR | Aspartate aminotransferase, mitochondrial | P00505 |
| AFAM_DADPDTFFAK | Afamin | P43652 |
| AFAM_FLVNLVK | Afamin | P43652 |
| AFAM_LPNNVLQEK | Afamin | P43652 |
| ALDOA_ALQASALK | Fructose-bisphosphate aldolase A | P04075 |
| ALDOA_QLLLTADDR | Fructose-bisphosphate aldolase A | P04075 |
| AMBP_AFIQLWAFDAVK | Protein AMBP | P02760 |
| AMBP_ETLLQDFR | Protein AMBP | P02760 |
| AMBP_FLYHK | Protein AMBP | P02760 |
| AMD_IPVDEEAFVIDFKPR | Peptidyl-glycine alpha-amidating monooxygenase | P19021 |
| AMD_IVQFSPSGK | Peptidyl-glycine alpha-amidating monooxygenase | P19021 |
| AMD_NGQWTLIGR | Peptidyl-glycine alpha-amidating monooxygenase | P19021 |
| APLP2_HYQHVLAVDPEK | Amyloid-like protein 2 | P05067 |
| APLP2_WYFDLSK | Amyloid-like protein 2 | P05067 |
| APOB_IAELSATAQEIIK | Apolipoprotein B-100 | P04114 |
| APOB_SVSLPSLDPASAK | Apolipoprotein B-100 | P04114 |
| APOB_TGISPLALIK | Apolipoprotein B-100 | P04114 |
| APOD_VLNQELR | Apolipoprotein D | P05090 |
| APOE_AATVGSLAGQPLQER | Apolipoprotein E | P02649 |
| APOE_CLAVYQAGAR | Apolipoprotein E | P02649 |
| APOE_LAVYQAGAR | Apolipoprotein E | P02649 |
| APOE_LGADMEDVR | Apolipoprotein E | P02649 |
| APOE_LGPLVEQGR | Apolipoprotein E | P02649 |
| B2MG_VEHSDLSFSK | Beta-2-microglobulin | P61769 |
| B2MG_VNHVTLSQPK | Beta-2-microglobulin | P61769 |
| B3GN1_EPGEFALLR | N-acetyllactosaminide beta-1,3-N-acetylglucosaminyltransferase | Q9NY97 |
| B3GN1_TALASGGVLDASGDYR | N-acetyllactosaminide beta-1,3-N-acetylglucosaminyltransferase | Q9NY97 |
| B3GN1_YEAAVPDPR | N-acetyllactosaminide beta-1,3-N-acetylglucosaminyltransferase | Q9NY97 |
| BACE1_SIVDSGTTNLR | Beta-secretase 1 | P56817 |
| BASP1_ETPAATEAPSSTPK | Brain acid soluble protein 1 | P80723 |
| BTD_LSSGLVTAALYGR | Biotinidase | P43251 |
| BTD_SHLIIAQVAK | Biotinidase | P43251 |
| C1QB_LEQGENVFLQATDK | Complement C1q subcomponent subunit B | P02746 |
| C1QB_VPGLYYFTYHASSR | Complement C1q subcomponent subunit B | P02746 |
| CA2D1_FVVTDGGITR | Voltage-dependent calcium channel subunit alpha-2/delta-1 | P54289 |
| CA2D1_IKPVFIEDANFGR | Voltage-dependent calcium channel subunit alpha-2/delta-1 | P54289 |
| CA2D1_TASGVNQLVDIYEK | Voltage-dependent calcium channel subunit alpha-2/delta-1 | P54289 |
| CAD13_DIQGSLQDIFK | Cadherin-13 | P55290 |
| CAD13_INENTGSVSVTR | Cadherin-13 | P55290 |
| CAD13_YEVSSPYFK | Cadherin-13 | P55290 |
| CADM3_EGSVPPLK | Cell adhesion molecule 3 | Q8N126 |
| CADM3_GNPVPQQYLWEK | Cell adhesion molecule 3 | Q8N126 |
| CADM3_SLVTVLGIPQKPIITGYK | Cell adhesion molecule 3 | Q8N126 |
| CAH1_VLDALQAIK | Carbonic anhydrase 1 | P00915 |
| CAH1_YSSLAEAASK | Carbonic anhydrase 1 | P00915 |
| CATA_LFAYPDTHR | Catalase | P04040 |
| CATD_LVDQNIFSFYLSR | Cathepsin D | P07339 |
| CATD_VSTLPAITLK | Cathepsin D | P07339 |
| CATD_YSQAVPAVTEGPIPEVLK | Cathepsin D | P07339 |
| CATL1_VFQEPLFYEAPR | Cathepsin L1 | P07711 |
| CCKN_AHLGALLAR | Cholecystokinin | P06307 |
| CCKN_NLQNLDPSHR | Cholecystokinin | P06307 |
| CD14_AFPALTSLDLSDNPGLGER | Monocyte differentiation antigen CD14 | P08571 |
| CD14_FPAIQNLALR | Monocyte differentiation antigen CD14 | P08571 |
| CD14_SWLAELQQWLKPGLK | Monocyte differentiation antigen CD14 | P08571 |
| CD59_AGLQVYNK | CD59 glycoprotein | P13987 |
| CERU_IYHSHIDAPK | Ceruloplasmin | P00450 |
| CERU_NNEGTYYSPNYNPQSR | Ceruloplasmin | P00450 |
| CFAB_DAQYAPGYDK | Complement factor B | P00751 |
| CFAB_VSEADSSNADWVTK | Complement factor B | P00751 |
| CFAB_YGLVTYATYPK | Complement factor B | P00751 |
| CH3L1_ILGQQVPYATK | Chitinase-3-like protein 1 | P36222 |
| CH3L1_SFTLASSETGVGAPISGPGIPGR | Chitinase-3-like protein 1 | P36222 |
| CH3L1_VTIDSSYDIAK | Chitinase-3-like protein 1 | P36222 |
| CLUS_IDSLLENDR | Clusterin | P10909 |
| CLUS_SGSGLVGR | Clusterin | P10909 |
| CLUS_VTTVASHTSDSDVPSGVTEVVVK | Clusterin | P10909 |
| CMGA_EDSLEAGLPLQVR | Chromogranin-A | P10645 |
| CMGA_SEALAVDGAGKPGAEEAQDPEGK | Chromogranin-A | P10645 |
| CMGA_SGEATDGARPQALPEPMQESK | Chromogranin-A | P10645 |
| CMGA_SGELEQEEER | Chromogranin-A | P10645 |
| CMGA_YPGPQAEGDSEGLSQGLVDR | Chromogranin-A | P10645 |
| CNDP1_ALEQDLPVNIK | Beta-Ala-His dipeptidase | Q96KN2 |
| CNDP1_VFQYIDLHQDEFVQTLK | Beta-Ala-His dipeptidase | Q96KN2 |
| CNDP1_WNYIEGTK | Beta-Ala-His dipeptidase | Q96KN2 |
| CNTN1_DGEYVVEVR | Contactin-1 | Q12860 |
| CNTN1_TTKPYPADIVVQFK | Contactin-1 | Q12860 |
| CNTN2_IIVQAQPEWLK | Contactin-2 | Q02246 |
| CNTN2_TTGPGGDGIPAEVHIVR | Contactin-2 | Q02246 |
| CNTN2_VIASNILGTGEPSGPSSK | Contactin-2 | Q02246 |
| CNTP2_HELQHPIIAR | Contactin-associated protein-like 2 | Q9UHC6 |
| CNTP2_VDNAPDQQNSHPDLAQEEIR | Contactin-associated protein-like 2 | Q9UHC6 |
| CNTP2_YSSSDWVTQYR | Contactin-associated protein-like 2 | Q9UHC6 |
| CO2_DFHINLFR | Complement C2 | P06681 |
| CO2_HAIILLTDGK | Complement C2 | P06681 |
| CO2_SSGQWQTPGATR | Complement C2 | P06681 |
| CO3_IHWESASLLR | Complement C3 | P01024 |
| CO3_LSINTHPSQKPLSITVR | Complement C3 | P01024 |
| CO3_TELRPGETLNVNFLLR | Complement C3 | P01024 |
| CO3_TGLQEVEVK | Complement C3 | P01024 |
| CO3_VPVAVQGEDTVQSLTQGDGVAK | Complement C3 | P01024 |
| CO4A_DHAVDLIQK | Complement C4-A | P0C0L4 |
| CO4A_GSFEFPVGDAVSK | Complement C4-A | P0C0L4 |
| CO4A_LGQYASPTAK | Complement C4-A | P0C0L4 |
| CO4A_NVNFQK | Complement C4-A | P0C0L4 |
| CO4A_VLSLAQEQVGGSPEK | Complement C4-A | P0C0L4 |
| CO4A_VTASDPLDTLGSEGALSPGGVASLLR | Complement C4-A | P0C0L4 |
| CO5_DINYVNPVIK | Complement C5 | P01031 |
| CO5_TLLPVSKPEIR | Complement C5 | P01031 |
| CO5_VFQFLEK | Complement C5 | P01031 |
| CO6_ALNHLPLEYNSALYSR | Complement component C6 | P13671 |
| CO6_SEYGAALAWEK | Complement component C6 | P13671 |
| CO8B_IPGIFELGISSQSDR | Complement component C8 beta chain | P07358 |
| CO8B_SDLEVAHYK | Complement component C8 beta chain | P07358 |
| CO8B_YEFILK | Complement component C8 beta chain | P07358 |
| COCH_GVISNSGGPVR | Cochlin | O43405 |
| CRP_ESDTSYVSLK | C-reactive protein | P02741 |
| CSTN1_GNLAGLTLR | Calsyntenin-1 | O94985 |
| CSTN1_IHGQNVPFDAVVVDK | Calsyntenin-1 | O94985 |
| CSTN1_IPDGVVSVSPK | Calsyntenin-1 | O94985 |
| CSTN3_ATGEGLIR | Calsyntenin-3 | Q9BQT9 |
| CSTN3_ESLLLDTTSLQQR | Calsyntenin-3 | Q9BQT9 |
| CUTA_TQSSLVPALTDFVR | Protein CutA | O60888 |
| CYTC_ALDFAVGEYNK | Cystatin-C | P01034 |
| DAG1_GVHYISVSATR | Dystroglycan | Q14118 |
| DAG1_LVPVVNNR | Dystroglycan | Q14118 |
| DAG1_VTIPTDLIASSGDIIK | Dystroglycan | Q14118 |
| DIAC_ATYIQNYR | Di-N-acetylchitobiase | Q01459 |
| ENOG_GNPTVEVDLYTAK | Gamma-enolase | P09104 |
| ENOG_LGAEVYHTLK | Gamma-enolase | P09104 |
| ENPP2_SYPEILTLK | Ectonucleotide pyrophosphatase/phosphodiesterase family member 2 | Q13822 |
| ENPP2_WWGGQPLWITATK | Ectonucleotide pyrophosphatase/phosphodiesterase family member 2 | Q13822 |
| EXTL2_VIVVWNNIGEK | Exostosin-like 2 | Q9UBQ6 |
| FABPH_SIVTLDGGK | Fatty acid-binding protein, heart | P05413 |
| FABPH_SLGVGFATR | Fatty acid-binding protein, heart | P05413 |
| FAM3C_GINVALANGK | Protein FAM3C | Q92520 |
| FAM3C_SALDTAAR | Protein FAM3C | Q92520 |
| FAM3C_SPFEQHIK | Protein FAM3C | Q92520 |
| FAM3C_TGEVLDTK | Protein FAM3C | Q92520 |
| FBLN1_AITPPHPASQANIIFDITEGNLR | Fibulin-1 | P23142 |
| FBLN1_IIEVEEEQEDPYLNDR | Fibulin-1 | P23142 |
| FBLN1_TGYYFDGISR | Fibulin-1 | P23142 |
| FBLN3_IPSNPSHR | EGF-containing fibulin-like extracellular matrix protein 1 | Q12805 |
| FBLN3_LTIIVGPFSF | EGF-containing fibulin-like extracellular matrix protein 1 | Q12805 |
| FBLN3_SGNENGEFYLR | EGF-containing fibulin-like extracellular matrix protein 1 | Q12805 |
| FETUA_AHYDLR | Alpha-2-HS-glycoprotein | P02765 |
| FETUA_FSVVYAK | Alpha-2-HS-glycoprotein | P02765 |
| FETUA_HTLNQIDEVK | Alpha-2-HS-glycoprotein | P02765 |
| FMOD_YLPFVPSR | Fibromodulin | Q06828 |
| GFAP_ALAAELNQLR | Glial fibrillary acidic protein | P14136 |
| GOLM1_DQLVIPDGQEEEQEAAGEGR | Golgi membrane protein 1 | Q8NBJ4 |
| GOLM1_QQLQALSEPQPR | Golgi membrane protein 1 | Q8NBJ4 |
| GRIA4_EYPGSETPPK | Glutamate receptor 4 | P48058 |
| GRIA4_LQNILEQIVSVGK | Glutamate receptor 4 | P48058 |
| GRIA4_NTDQEYTAFR | Glutamate receptor 4 | P48058 |
| HBA_FLASVSTVLTSK | Hemoglobin subunit alpha | P69905 |
| HBA_TYFPHFDLSHGSAQVK | Hemoglobin subunit alpha | P69905 |
| HBA_VGAHAGEYGAEALER | Hemoglobin subunit alpha | P69905 |
| HBB_EFTPPVQAAYQK | Hemoglobin subunit beta | P68871 |
| HBB_SAVTALWGK | Hemoglobin subunit beta | P68871 |
| HBB_VNVDEVGGEALGR | Hemoglobin subunit beta | P68871 |
| HEMO_NFPSPVDAAFR | Hemopexin | P02790 |
| HEMO_QGHNSVFLIK | Hemopexin | P02790 |
| HEMO_SGAQATWTELPWPHEK | Hemopexin | P02790 |
| I18BP_LWEGSTSR | Interleukin-18-binding protein | O95998 |
| IBP2_HGLYNLK | Insulin-like growth factor-binding protein 2 | P18065 |
| IBP2_LIQGAPTIR | Insulin-like growth factor-binding protein 2 | P18065 |
| IGSF8_DTQFSYAVFK | Immunoglobulin superfamily member 8 | Q969P0 |
| IGSF8_LQGDAVVLK | Immunoglobulin superfamily member 8 | Q969P0 |
| IGSF8_VVAGEVQVQR | Immunoglobulin superfamily member 8 | Q969P0 |
| ITIH1_EVAFDLEIPK | Inter-alpha-trypsin inhibitor heavy chain H1 | P19827 |
| ITIH1_QYYEGSEIVVAGR | Inter-alpha-trypsin inhibitor heavy chain H1 | P19827 |
| ITIH5_SYLEITPSR | Inter-alpha-trypsin inhibitor heavy chain H5 | Q86UX2 |
| KAIN_FYYLIASETPGK | Kallistatin | P29622 |
| KAIN_LGFTDLFSK | Kallistatin | P29622 |
| KAIN_VGSALFLSHNLK | Kallistatin | P29622 |
| KAIN_WADLSGITK | Kallistatin | P29622 |
| KLK10_ALQLPYR | Kallikrein-10 | O43240 |
| KLK11_LPHTLR | Kallikrein-11 | Q9UBX7 |
| KLK6_ESSQEQSSVVR | Kallikrein-6 | Q92876 |
| KLK6_LSELIQPLPLER | Kallikrein-6 | Q92876 |
| KLK6_YTNWIQK | Kallikrein-6 | Q92876 |
| KNG1_DIPTNSPELEETLTHTITK | Kininogen-1 | P01042 |
| KNG1_QVVAGLNFR | Kininogen-1 | P01042 |
| KNG1_TVGSDTFYSFK | Kininogen-1 | P01042 |
| KPYM_LDIDSPPITAR | Pyruvate kinase isozymes M1/M2 | P14618 |
| L1CAM_AQLLVVGSPGPVPR | Neural cell adhesion molecule L1 | P32004 |
| L1CAM_LVLSDLHLLTQSQVR | Neural cell adhesion molecule L1 | P32004 |
| L1CAM_WRPVDLAQVK | Neural cell adhesion molecule L1 | P32004 |
| LAMB2_AQGIAQGAIR | Laminin subunit beta-2 | Q61292 |
| LPHN1_LVVSQLNPYTLR | Latrophilin-1 | O94910 |
| LPHN1_SGETVINTANYHDTSPYR | Latrophilin-1 | O94910 |
| LRC4B_HLEILQLSK | Leucine-rich repeat-containing protein 4B | Q9NT99 |
| LRC4B_LTTVPTQAFEYLSK | Leucine-rich repeat-containing protein 4B | Q9NT99 |
| LTBP2_EQDAPVAGLQPVER | Latent-transforming growth factor beta-binding protein 2 | Q14767 |
| MIME_ESAYLYAR | Mimecan | P20774 |
| MIME_ETVIIPNEK | Mimecan | P20774 |
| MIME_LEGNPIVLGK | Mimecan | P20774 |
| MOG_VVHLYR | Myelin-oligodendrocyte glycoprotein | Q16653 |
| MUC18_EVTVPVFYPTEK | Cell surface glycoprotein MUC18 | P43121 |
| MUC18_GATLALTQVTPQDER | Cell surface glycoprotein MUC18 | P43121 |
| NBL1_LALFPDK | Neuroblastoma suppressor of tumorigenicity 1 | P41271 |
| NCAM1_AGEQDATIHLK | Neural cell adhesion molecule 1 | P13591 |
| NCAM1_GLGEISAASEFK | Neural cell adhesion molecule 1 | P13591 |
| NCAM2_ASGSPEPAISWFR | Neural cell adhesion molecule 2 | O15394 |
| NCAM2_IIELSQTTAK | Neural cell adhesion molecule 2 | O15394 |
| NCAN_APVLELEK | Neurocan core protein | O14594 |
| NCAN_LSSAIIAAPR | Neurocan core protein | O14594 |
| NEGR1_SSIIFAGGDK | Neuronal growth regulator 1 | Q7Z3B1 |
| NEGR1_VVVNFAPTIQEIK | Neuronal growth regulator 1 | Q7Z3B1 |
| NEGR1_WSVDPR | Neuronal growth regulator 1 | Q7Z3B1 |
| NELL2_AFLFQDTPR | Protein kinase C-binding protein NELL2 | Q99435 |
| NELL2_FTGSSWIK | Protein kinase C-binding protein NELL2 | Q99435 |
| NELL2_SALAYVDGK | Protein kinase C-binding protein NELL2 | Q99435 |
| NEO1_DVVASLVSTR | Neogenin | Q92859 |
| NEUS_ALGITEIFIK | Neuroserpin | Q99574 |
| NEUS_QEVPLATLEPLVK | Neuroserpin | Q99574 |
| NGF_SAPAAAIAAR | Beta-nerve growth factor | P01138 |
| NICA_ALADVATVLGR | Nicastrin | Q92542 |
| NICA_APDVTTLPR | Nicastrin | Q92542 |
| NPTX1_FQLTFPLR | Neuronal pentraxin-1 | Q15818 |
| NPTX1_LENLEQYSR | Neuronal pentraxin-1 | Q15818 |
| NPTX2_LESLEHQLR | Neuronal pentraxin-2 | P47972 |
| NPTX2_TESTLNALLQR | Neuronal pentraxin-2 | P47972 |
| NPTXR_ELDVLQGR | Neuronal pentraxin receptor | O95502 |
| NPTXR_LVEAFGGATK | Neuronal pentraxin receptor | O95502 |
| NRCAM_SLPSEASEQYLTK | Neuronal cell adhesion molecule | Q92823 |
| NRCAM_VFNTPEGVPSAPSSLK | Neuronal cell adhesion molecule | Q92823 |
| NRCAM_YIVSGTPTFVPYLIK | Neuronal cell adhesion molecule | Q92823 |
| NRX1A_DLFIDGQSK | Neurexin-1 | Q9ULB1 |
| NRX1A_ITTQITAGAR | Neurexin-1 | Q9ULB1 |
| NRX1A_SDLYIGGVAK | Neurexin-1 | Q9ULB1 |
| NRX2A_AIVADPVTFK | Neurexin-2 | Q9P2S2 |
| NRX2A_LGERPPALLGSQGLR | Neurexin-2 | Q9P2S2 |
| NRX2A_LSALTLSTVK | Neurexin-2 | Q9P2S2 |
| NRX3A_IYGEVVFK | Neurexin-3 | Q9Y4C0 |
| NRX3A_SDLSFQFK | Neurexin-3 | Q9Y4C0 |
| OSTP_AIPVAQDLNAPSDWDSR | Osteopontin | P10451 |
| PCSK1_ALAHLLEAER | ProSAAS | Q9UHG2 |
| PCSK1_GEAAGAVQELAR | ProSAAS | Q9UHG2 |
| PCSK1_NSDPALGLDDDPDAPAAQLAR | ProSAAS | Q9UHG2 |
| PDYN_FLPSISTK | Proenkephalin-B | P01213 |
| PDYN_LSGSFLK | Proenkephalin-B | P01213 |
| PDYN_SVGEGPYSELAK | Proenkephalin-B | P01213 |
| PEDF_DTDTGALLFIGK | Pigment epithelium-derived factor | P36955 |
| PEDF_SSFVAPLEK | Pigment epithelium-derived factor | P36955 |
| PEDF_TVQAVLTVPK | Pigment epithelium-derived factor | P36955 |
| PGRP2_AGLLRPDYALLGHR | N-acetylmuramoyl-L-alanine amidase | Q96PD5 |
| PGRP2_TFTLLDPK | N-acetylmuramoyl-L-alanine amidase | Q96PD5 |
| PIMT_VQLVVGDGR | Protein-L-isoaspartate(D-aspartate) O-methyltransferase | P22061 |
| PLDX1_LYGPSEPHSR | Plexin domain-containing protein 1 | Q8IUK5 |
| PLMN_EAQLPVIENK | Plasminogen | P00747 |
| PLMN_HSIFTPETNPR | Plasminogen | P00747 |
| PLMN_LSSPAVITDK | Plasminogen | P00747 |
| PPN_VHQSPDGTLLIYNLR | Papilin | O95428 |
| PRDX1_DISLSDYK | Peroxiredoxin-1 | Q06830 |
| PRDX1_LVQAFQFTDK | Peroxiredoxin-1 | Q06830 |
| PRDX2_GLFIIDGK | Peroxiredoxin-2 | P32119 |
| PRDX2_IGKPAPDFK | Peroxiredoxin-2 | P32119 |
| PRDX3_HLSVNDLPVGR | Thioredoxin-dependent peroxide reductase, mitochondrial | P30048 |
| PRDX6_LIALSIDSVEDHLAWSK | Peroxiredoxin-6 | P30041 |
| PRDX6_LSILYPATTGR | Peroxiredoxin-6 | P30041 |
| PTGDS_AQGFTEDTIVFLPQTDK | Prostaglandin-H2 D-isomerase | P41222 |
| PTGDS_WFSAGLASNSSWLR | Prostaglandin-H2 D-isomerase | P41222 |
| PTPRN_AEAPALFSR | Receptor-type tyrosine-protein phosphatase-like N | Q16849 |
| PTPRN_LAAVLAGYGVELR | Receptor-type tyrosine-protein phosphatase-like N | Q16849 |
| PTPRN_SELEAQTGLQILQTGVGQR | Receptor-type tyrosine-protein phosphatase-like N | Q16849 |
| PVRL1_ITQVTWQK | Poliovirus receptor-related protein 1 | G3IGH2 |
| SCG1_GEAGAPGEEDIQGPTK | Secretogranin-1 | P05060 |
| SCG1_HLEEPGETQNAFLNER | Secretogranin-1 | P05060 |
| SCG1_NYLNYGEEGAPGK | Secretogranin-1 | P05060 |
| SCG1_SSQGGSLPSEEK | Secretogranin-1 | P05060 |
| SCG2_ALEYIENLR | Secretogranin-2 | P13521 |
| SCG2_IILEALR | Secretogranin-2 | P13521 |
| SCG2_VLEYLNQEK | Secretogranin-2 | P13521 |
| SCG3_ELSAERPLNEQIAEAEEDK | Secretogranin-3 | Q8WXD2 |
| SCG3_FQDDPDGLHQLDGTPLTAEDIVHK | Secretogranin-3 | Q8WXD2 |
| SCG3_LNVEDVDSTK | Secretogranin-3 | Q8WXD2 |
| SE6L1_ETGTPIWTSR | Seizure 6-like protein | Q9BYH1 |
| SE6L1_SPTNTISVYFR | Seizure 6-like protein | Q9BYH1 |
| SIAE_ELSNTAAYQSVR | Sialate O-acetylesterase | Q9HAT2 |
| SLIK1_SLPVDVFAGVSLSK | SLIT and NTRK-like protein 1 | Q96PX8 |
| SODC_GDGPVQGIINFEQK | Superoxide dismutase [Cu-Zn] | P00441 |
| SODC_HVGDLGNVTADK | Superoxide dismutase [Cu-Zn] | P00441 |
| SODC_TLVVHEK | Superoxide dismutase [Cu-Zn] | P00441 |
| SODE_AGLAASLAGPHSIVGR | Extracellular superoxide dismutase [Cu-Zn] | P08294 |
| SODE_AVVVHAGEDDLGR | Extracellular superoxide dismutase [Cu-Zn] | P08294 |
| SODE_VTGVVLFR | Extracellular superoxide dismutase [Cu-Zn] | P08294 |
| SORC1_TIAVYEEFR | VPS10 domain-containing receptor SorCS1 | Q8WY21 |
| SPON1_VTLSAAPPSYFR | Spondin-1 | Q9HCB6 |
| SPRL1_HIQETEWQSQEGK | SPARC-like protein 1 | Q14515 |
| SPRL1_HSASDDYFIPSQAFLEAER | SPARC-like protein 1 | Q14515 |
| SPRL1_VLTHSELAPLR | SPARC-like protein 1 | Q14515 |
| TGFB1_LLAPSDSPEWLSFDVTGVVR | Transforming growth factor beta-1 | P01137 |
| THRB_ETAASLLQAGYK | Prothrombin | P00734 |
| THRB_YGFYTHVFR | Prothrombin | P00734 |
| TIMP1_GFQALGDAADIR | Metalloproteinase inhibitor 1 | P01033 |
| TIMP1_SEEFLIAGK | Metalloproteinase inhibitor 1 | P01033 |
| TNR21_ASNLIGTYR | Tumor necrosis factor receptor superfamily member 21 | O75509 |
| TRFM_ADTDGGLIFR | Melanotransferrin | P08582 |
| TTHY_TSESGELHGLTTEEEFVEGIYK | Transthyretin | P02766 |
| TTHY_VEIDTK | Transthyretin | P02766 |
| UBB_ESTLHLVLR | Polyubiquitin-B | P0CG47 |
| UBB_TITLEVEPSDTIENVK | Polyubiquitin-B | P0CG47 |
| UBB_TLSDYNIQK | Polyubiquitin-B | P0CG47 |
| VASN_NLHDLDVSDNQLER | Vasorin | Q6EMK4 |
| VASN_SLTLGIEPVSPTSLR | Vasorin | Q6EMK4 |
| VASN_YLQGSSVQLR | Vasorin | Q6EMK4 |
| VGF_AYQGVAAPFPK | Neurosecretory protein VGF | O15240 |
| VGF_NSEPQDEGELFQGVDPR | Neurosecretory protein VGF | O15240 |
| VGF_THLGEALAPLSK | Neurosecretory protein VGF | O15240 |
| VTDB_EFSHLGK | Vitamin D-binding protein | P02774 |
| VTDB_HLSLLTTLSNR | Vitamin D-binding protein | P02774 |
| VTDB_VPTADLEDVLPLAEDITNILSK | Vitamin D-binding protein | P02774 |
