# Supplementary material for: A multivariate predictive modeling approach reveals a novel CSF peptide signature for both Alzheimer's Disease state classification and for predicting future disease progression
Source: PLoS One. 2017 Aug 3;12(8):e0182098. doi: 10.1371/journal.pone.0182098 (PMC5542644; doi:10.1371/journal.pone.0182098)
Supplement: S2 Table — Coefficients for the signature peptides are given. Caution should be exercised in interpreting these coefficients: 1. Lower coefficient does not imply less importance because the scales of the intensity values are different between the peptides, and 2. These values should not be applied directly in practice unless the same exact assay platform is used. (DOCX) [file pone.0182098.s002.docx]

S2 Table. Signature Coefficients. Coefficients for the signature peptides are given. Caution should be exercised in interpreting these coefficients: 1. Lower coefficient does not imply less importance because the scales of the intensity values are different between the peptides, and 2. These values should not be applied directly in practice unless the same exact assay platform is used.

| **Signature peptides** | **Coefficient** |
| --- | --- |
| FABPH.SIVTLDGGK | 2.2 |
| FABPH.SLGVGFATR | 1.9 |
| NPTXR.ELDVLQGR | -1.7 |
| TTHY.TSESGELHGLTTEEEFVEGIYK | 1.6 |
| ALDOA.ALQASALK | 1.4 |
| DAG1.GVHYISVSATR | -1.3 |
| PTPRN.AEAPALFSR | -1.1 |
| AMD.IVQFSPSGK | -0.8 |
| PIMT.VQLVVGDGR | -0.8 |
| CA2D1.FVVTDGGITR | -0.7 |
| KLK11.LPHTLR | 0.6 |
| CATD.LVDQNIFSFYLSR | 0.4 |
| PRDX1.LVQAFQFTDK | 0.3 |
| APOE.LGADMEDVR | 0.1 |
| PTPRN.SELEAQTGLQILQTGVGQR | -0.1 |
| VGF.NSEPQDEGELFQGVDPR | -0.1 |
